# Impact of addictive comorbidity on bipolar disorder type I

**DOI:** 10.1192/j.eurpsy.2024.985

**Published:** 2024-08-27

**Authors:** A. Adouni, Y. Zgueb, F. Ben Othman, I. Bouguerra, R. Jomli

**Affiliations:** psychiatry department A, Razi hospital, Manouba, Tunisia

## Abstract

**Introduction:**

Among all mental pathologies, bipolar disorder (BD) is the one in which addictive comorbidity is most frequent.

Recent studies suggest that this comorbidity has harmful consequences, threatening patients’ quality of life.

**Objectives:**

Describe addictive comorbidity and determine its prevalence in a population of patients with BD I.

Study the impact of addictive comorbidity on the evolution of BD I.

**Methods:**

A cross-sectional, comparative study was conducted over a six-month period in the after-care unit of psychiatric wards at Razi Hospital, including patients treated for BD I according to DSM 5 criteria and stable on treatment.

The study included two phases: first, sociodemographic, clinical and therapeutic characteristics were collected using a pre-established form. The CAGE, DUDIT and MARS scales, validated in Arabic, were then administered.

**Results:**

We included 100 patients (60 men and 40 women) with a mean age of 43.55 years.

Substance use disorder (SUD) was reported in 31% of our population; 22 alcohol users with a mean CAGE score of 1.23 (0-3), while psychoactive substance use was reported in 19 patients with a mean DUDIT score of 13.37 (0-28).

Forensic history was higher in the group of patients with comorbid SUD (p<0.001). Poor compliance with treatment and irregular follow-up were also significantly more associated with addictive behavior, respectively p=0.008 and p=0.048.

We found no association between SUD and suicidal behavior or evolutionary symptoms of the disorder.

**Conclusions:**

SUD are generally factors in the poor prognosis of BD. It is important to identify the determinants of this comorbidity, so that these risk factors can be appropriately targeted through appropriate therapeutic interventions and thus limit these negative consequences.

**Disclosure of Interest:**

None Declared

